# Association between diabetes and food insecurity in an urban setting in Angola: a case–control study

**DOI:** 10.1038/s41598-022-04888-7

**Published:** 2022-01-20

**Authors:** Claudia Robbiati, António Armando, Natália da Conceição, Giovanni Putoto, Francesco Cavallin

**Affiliations:** 1 Doctors with Africa, Luanda, Angola; 2 National Directory of Public Health, Ministry of Health of Angola, Luanda, Angola; 3 Doctors with Africa, Padua, Italy; 4 Independent Statistician, Solagna, Italy

**Keywords:** Type 2 diabetes, Preventive medicine

## Abstract

Diabetes is common in urban settings in Sub-Saharan Africa. Household food insecurity has been suggested to increase the chance of developing diabetes among adults. The relationship between diabetes and food insecurity has not been explored in Angolan urban settings so far. This case–control (1:2) study investigated the association between diabetes and food insecurity among adults attending six healthcare facilities in Luanda (Angola) between April 2019 and September 2019. All subjects with fasting blood glucose (FBG) levels ≥ 126 mg/dl were included as cases. For each case, the next two subjects with FBG levels < 110 mg/dl were included as controls, to warrant the achievement of the set 1:2 ratio. Food insecurity was assessed using the Food Insecurity Experience Scale (FIES). A total of 663 participants (221 cases and 442 controls) were enrolled in the study. Median FIES raw score was 7 (IQR 1–8) in cases and 5 (IQR 2–8) in controls (*p* = 0.09). The distribution of FIES levels (0–3; 4–6; 7–8) was different between cases and controls (*p* < 0.0001), with highest FIES scores (7–8) recorded in 53.0% of cases and 38.2% of controls. Our findings revealed an association between diabetes and severe food insecurity among adults attending healthcare facilities in the capital city of Angola.

## Introduction

Diabetes is an increasing public health burden in Sub-Saharan Africa (SSA). In 2019, 4.7% of adults aged 20–79 years were estimated to be living with diabetes in SSA, and this percentage is expected to rise to 5.1% by 2030^1^.

Diabetes is common in urban settings in SSA, where lifestyle changes such as unhealthy eating behaviours and adoption of a sedentary lifestyle may lead to obesity and development of non-communicable diseases (NCDs)^2^. As a matter of fact, a healthy diet is fundamental for diabetes prevention and management, but in low-resource settings adherence to an adequate dietary regimen may be hampered by food insecurity^3^.

According to the Food and Agriculture Organization (FAO), food security is defined as “everyone, at all times, having physical and economic access to sufficient, safe, nutritious food that meets their dietary needs for an active and healthy life”^4^. Food security has been recognized as a pivotal determinant of health and its role in supporting physical and mental health of individuals is unquestionable^5^. Hence, several indicators have been proposed to measure food insecurity^6,7^.

A recent systematic review in high and middle-income countries supported the hypothesis that household food insecurity may increase the chance of developing diabetes among adults, and suggested the following mechanism: food insecure individuals usually adopt unhealthy eating behaviours such as consuming processed foods which are inexpensive and easily accessible^8^. This can lead to an increased total energy intake, accumulation of visceral fat, and subsequent development of chronic disease like diabetes.

Food security is related to all the United Nations Sustainable Development Goals (SDGs) to be reached by 2030, and particularly to #3 (good health) and #11 (sustainable cities)^9^. As a matter of fact, the process of urbanization may hamper the access to food for poor and vulnerable people, which leads to a rise in hunger and fatalities, and limits the development of the community^10^.

Luanda is the capital city of Angola and is experiencing an epidemiological transition and a double burden of communicable and non-communicable diseases (as common in SSA urban settings) due to changes in lifestyle, diet and physical activity^11^. A recent cross-sectional study showed a diabetes prevalence of 12% among adults reaching health centres in Luanda, with lifestyle factors such as low consumption of vegetables, high consumption of free-sugars food/beverages and sedentarism associated with diabetes^12^. Moreover, in Luanda as well as other SSA settings, the burden of infectious diseases like tuberculosis, contributes to the burden of diabetes^13^. Food insecurity is also related to tuberculosis resulting in suboptimal adherence to treatment and consequent poor outcomes^14^.

Dedicated literature offers few studies on the relationship between food insecurity and diabetes in SSA. A study in Kenya highlighted the high prevalence of food insecurity amongst patients with diabetes in a resource-constrained setting^15^. A study in South Africa suggested that food insecurity hampers glycemic control in patients with diabetes, therefore primary care settings should promote early identification and management of food insecurity in diabetic patients^16^. Finally, food insecurity and medical insecurity were shown to be critical for diabetes patients' clinical presentations and prognoses in Ethiopia^17^. To our knowledge, the relationship between diabetes and food insecurity has not been explored in Angolan urban settings so far. Therefore, this study aimed at investigating the association between diabetes and food insecurity among adults accessing healthcare facilities in the capital city Luanda.

## Methods

### Study design

This was a case–control (1:2) study that investigated the association between diabetes and food insecurity among people attending healthcare facilities in Luanda (Angola). The study was part of a larger project on the prevention and the management of diabetes in Luanda.

### Setting

The study was carried out in Luanda, the capital city of Angola. According to the last edition of the 2014 census^18^, about 6,542,944 inhabitants live in the capital. Six health centres, from six different urban districts, were randomly included in the study. Subjects reaching the six health centers between April and September 2019 were appraised for inclusion criteria to be enrolled in the study.

### Participants

Eligible subjects were adults (≥ 18 years) of both sexes attending the six health centers between April 2019 and September 2019. Pregnant women, people not fasting for at least 8 h and people with a previous diagnosis of diabetes were not eligible for the study.

Eligible subjects had their fasting blood glucose (FBG) measured by professional nurses using a glucometer (Infopia, South Korea). All subjects with FBG levels ≥ 126 mg/dl were included as cases^19^. For each case, the next two subjects with FBG levels < 110 mg/dl were included as controls, to warrant the achievement of the set 1:2 ratio.

### Data collection

Research data were collected by professional nurses using a case-report form. Data included demographics (age and sex), clinical parameters (weight, body mass index BMI, waist circumference, systolic blood pressure SBP, diastolic blood pressure DBP and heart rate) and food insecurity levels.

**Figure 1:**
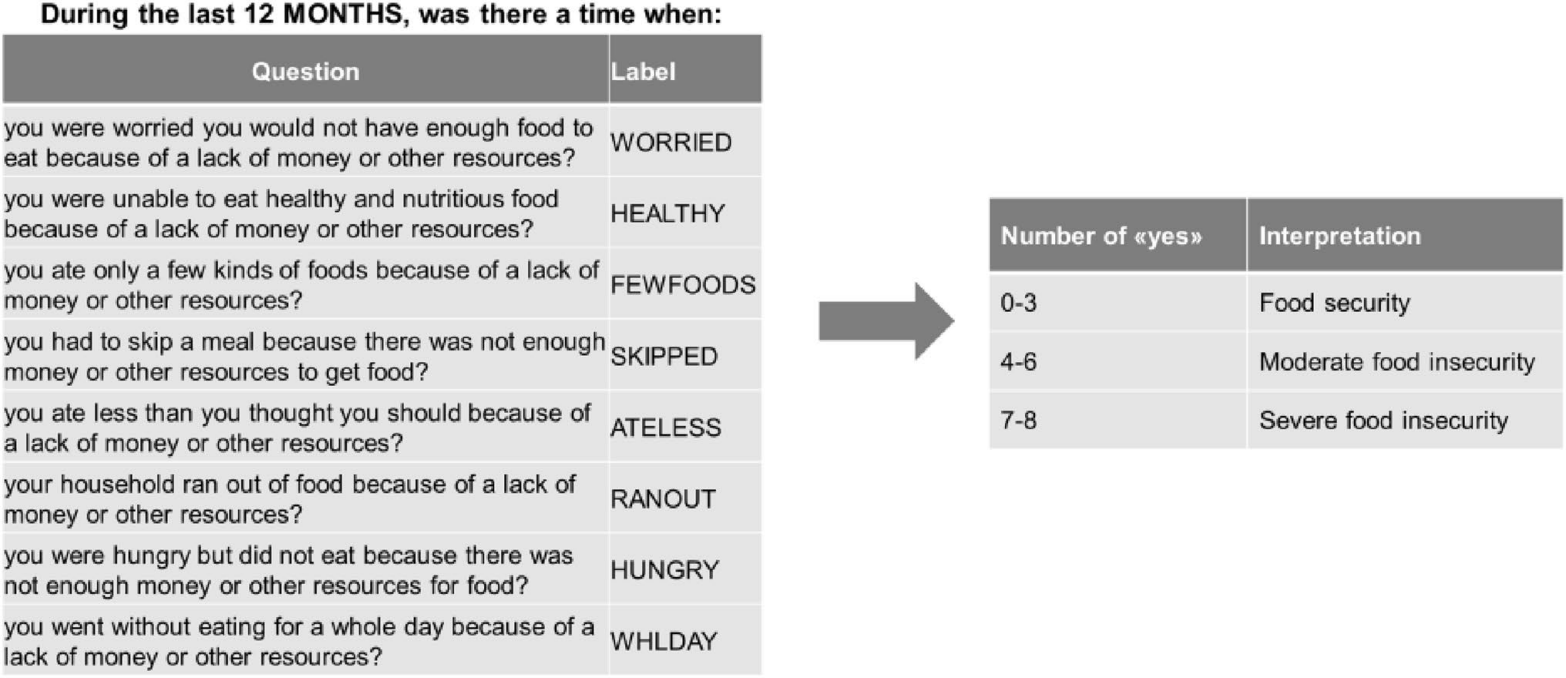
Food Insecurity Experience Scale (FIES): description and interpretation.

Food insecurity was assessed using the Food Insecurity Experience Scale (FIES), developed by the Food and Agriculture Organization of the United Nations (FAO)^20^. The FIES is one of the indicators to track progress toward reaching the Sustainable Development Goals (SDGs), particularly for goal 2.1, which aims to end hunger and ensure access to food by all people to safe, nutritious and sufficient food all year. The FIES consists of eight questions about respondent’s access to food of adequate quality and quantity over the last 12 months (Fig. 1).

Participants were classified into three food insecurity levels according to the respondents scores. Scores 0–3 indicate food security, scores 4–6 indicate moderate food insecurity and scores 7–8 indicate severe food insecurity^20,21^.

All data were collected by a professional nurse before participants went into the doctor’s consultation room. Data collection was coordinated and supervised by a research assistant.

### Statistical analysis

Categorical data were summarized as frequency and percentage, while continuous data as median and interquartile range (IQR). Categorical data were compared between two groups using Chi Square test, while continuous data using Mann–Whitney test. Severe food insecurity (SFI) and moderate-severe food insecurity (MSFI) were compared between cases and controls, and effect sizes were reported as odds ratio (OR) with 95% confidence interval^20,21^. Single items were presented with descriptive purpose and not used in single-item comparisons^21^. All tests were 2-sided and a p-value less than 0.05 was considered statistically significant. Statistical analysis was performed using R 4.0 (R Foundation for Statistical Computing, Vienna, Austria)^22^.

### Ethics

This study was approved by the National Public Health Directorate of the Ministry of Health of Angola and by the Ethics Committee of the Ministry of Health of Angola (number 21/2018). Each participant signed a full informed consent form. All methods were performed in accordance with the relevant guidelines and regulations. The study used anonymized data and no identifiable data were collected.

## Results

A total of 663 participants (221 cases and 442 controls) were enrolled in the study from April to September 2019. There were 216 males and 447 females, with a median age 38 years (IQR 28–51; range 18–88). Participant characteristics are shown in Table 1. Diabetes was associated with older age (*p* < 0.0001), and higher BMI (*p* = 0.0004), waist circumference (*p* < 0.0001), heart rate (*p* = 0.002) and systolic and diastolic blood pressure (*p* < 0.0001).

**Table 1:** Participant characteristics.

	Cases (diabetes)	Controls (no diabetes)	p-value
N	221	442	–
Age, years^ab^	46 (35–57)	35 (27–46)	< 0.0001
Males	67 (30.3)	149 (33.7)	0.43
Weight, kg^ac^	68 (56–79)	65 (56–74)	0.09
BMI, kg/m^2ad^	24.9 (21.4–29.0)	23.1 (20.1–26.6)	0.0004
Waist circumference, cm^ae^	86 (77–98)	77 (64–88)	< 0.0001
Systolic blood pressure, mmHg^af^	130 (120–154)	123 (114–136)	< 0.0001
Diastolic blood pressure, mmHg^ag^	80 (71–90)	76 (67–83)	< 0.0001
Heart rate, bpm^ah^	77 (68–88)	73 (65–84)	0.002

Data expressed as n (%) or ^a^median (IQR). Data not available in ^b^2, ^c^1, ^d^10, ^e^22, ^f^3, ^g^9 and ^h^17 participants.

**Figure 2:**
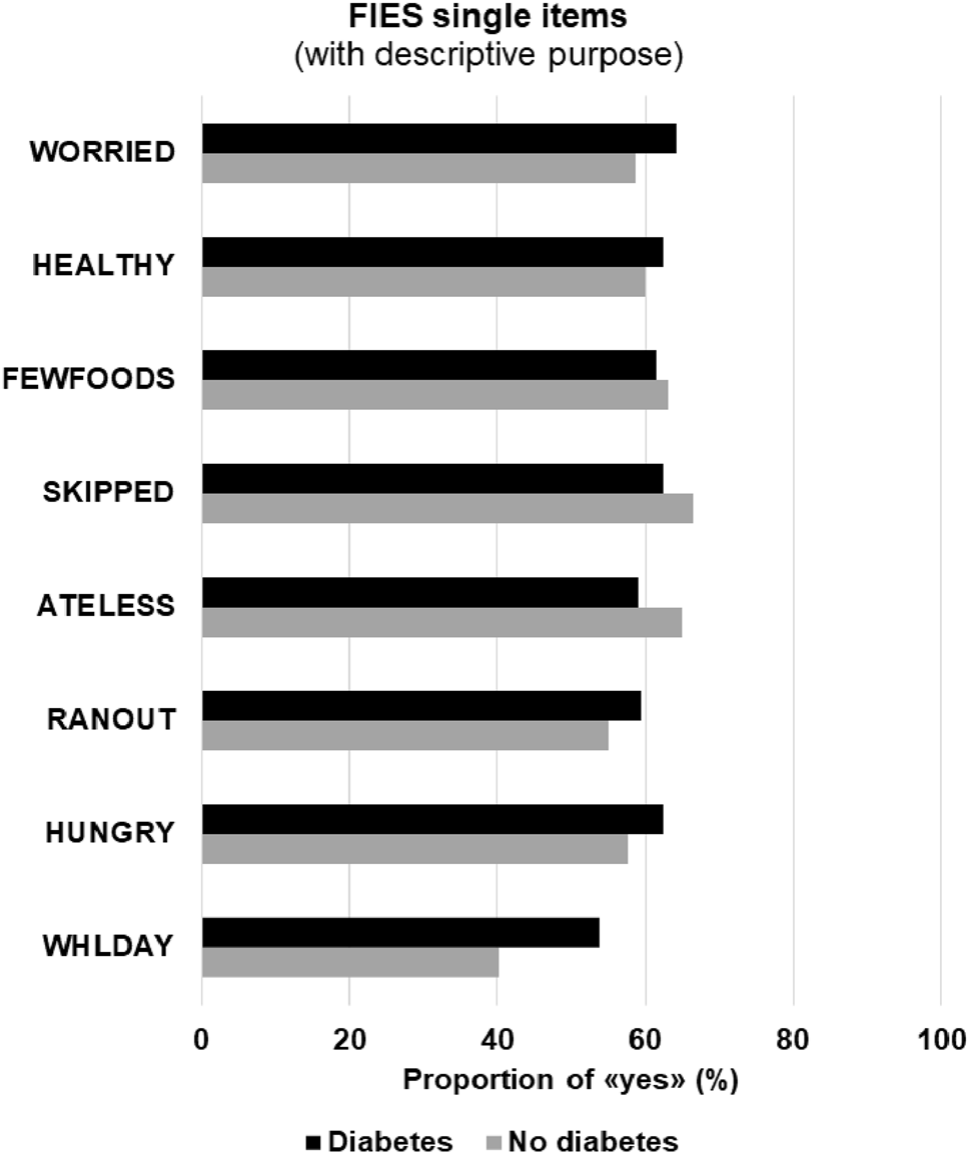
Single items of FIES in cases and controls.

Food security and food insecurity were calculated according to FIES in 215 cases and 432 controls (Table 2), while the information was incomplete in the other 16 participants. MSFI was found in 135/215 cases (62.8%) and 276/432 controls (63.9%) (OR 0.95, 95% CI 0.68 to 1.34; *p* = 0.78). SFI was found in 114/215 cases (53.0%) and 165/432 controls (38.2%) (OR 1.83, 95% CI 1.31 to 2.54; *p* = 0.0004). Figure 2 displays occurrence of affirmative answers to single items with descriptive purpose.

**Table 2:** Food insecurity according to FIES among cases and controls.

	Cases (diabetes)	Controls (no diabetes)
N subjects with available FIES	215/221	432/442
Food security	80 (37.2)	156 (36.1)
Moderate food insecurity	21 (9.8)	111 (25.7)
Severe food insecurity	114 (53.0)	165 (38.2)

## Discussion

Our findings revealed an association between diabetes and severe food insecurity among adults attending healthcare facilities in the capital city of Angola. To our knowledge, this is the first study on this topic in Angola and it contributes to the investigation of the relationship between diabetes and food insecurity in SSA. In particular, our investigation focused on an urban setting since many SSA countries have been facing a rise in NCDs due to diet and lifestyle changes associated with urbanization^11^. Moreover, food insecurity affects billions of urban poor in low resource settings and growing literature links food insecurity with adverse diabetes outcomes^8^.

SSA is currently home to 1.1 billion people and its population is predicted to reach 2.4 billion by 2050^23^. Hence, urbanization in SSA should be considered as an opportunity to develop food security strategies to prevent the rise of diabetes and NCDs^11^.

In Angolan urban settings, a paucity of studies have explored diabetes and its determinants, and no study investigated the role of food insecurity for diabetes outcomes so far. In the capital city Luanda, a recent study found that some diet and lifestyle factors (such as low consumption of vegetables, daily consumption of free-sugars foods and beverages, and time spent seated) were associated with diabetes and/or Impaired Fasting Glucose^12^. Of note, our data corroborated the previous association between diabetes and some demographic parameters (older age, high BMI and high blood pressure).

Mechanisms for the association between food insecurity and diabetes could be linked to the fact that food insecure people report skipping meals, eating more energy-dense foods and have a lower dietary quality, which is associated with obesity and the increased risk of developing diabetes^3^. Our study confirmed a higher proportion of severe food insecurity among diabetic adults in Angola. Unfortunately, our data did not allow to explore the mechanisms underlying this association. Of note, more diabetic participants reported to have skipped meals for a whole day, but the single items of the FIES should not be used for direct comparisons^18^.

Food insecurity is an important target for diabetes prevention and management since it is associated with poor glycaemic control, higher rates of complications and hospitalization, and poor adherence to treatment^24^. Therefore, addressing food insecurity may help tackle the burgeoning challenge of diabetes in SSA, where health systems are already overwhelmed by infectious diseases and are struggling to cope with the burden of non-communicable diseases^23^. Food insecurity screening among individuals with diabetes could help healthcare workers to identify patients’ difficulties in adhering to treatment and dietary recommendations^24^. Policies addressing food insecurity should be included into diabetes programs to promote the access to sufficient, safe and nutritious foods^25^. Poverty is one of the root causes of food insecurity, therefore local economy growth and food systems strengthening should be a priority in SSA. Strategies and policies to increase affordability and availability of healthy food like fruits and vegetables, should be promoted by the means of vouchers, investments in agriculture, incentives to healthy low-cost foods retailers and promotion of community initiatives, like urban gardens and farmers markets^3^.

The present study has some limitations that should be considered. First, the case–control design precludes any causal association between diabetes and food insecurity, which should be investigated with large prospective studies. Second, the participants were adults attending health facilities in Luanda, thus the generalization of the findings should be limited to similar settings (i.e. adults attending health facilities in urban areas in Sub-Saharan countries) and to newly diagnosed diabetic adults. In addition, sampling bias may have been introduced by the voluntary participation to the FBG measurement. Third, data on socio-economic status of the participants were not collected. Fourth, we relied only on point-of-care testing to classify diabetes, since other diagnostic tests were not available.

This study is a first attempt to explore the relationship between diabetes and food insecurity in an urban setting in Angola and it calls for further longitudinal studies to look into the pathways between diabetes and food insecurity. In future studies, uniformity of indicators to assess food insecurity levels should be adopted, for example by using the FIES at individual level, that is easy to perform and interpret. In addition, the mechanisms underlying the association between diabetes and food insecurity warrant further investigation.

## Conclusions

Our findings revealed an association between diabetes and severe food insecurity among adults attending healthcare facilities in the capital city of Angola. Further longitudinal studies are required to assess the pathway linking diabetes and food insecurity in this setting.

## Data Availability

The datasets used and/or analyzed during the current study are available from the corresponding author on reasonable request.
